# Efficient population representation with more genetic markers increases performance of a steelhead (Oncorhynchus mykiss) genetic stock identification baseline

**DOI:** 10.1111/eva.13610

**Published:** 2023-12-26

**Authors:** John S. Hargrove, Thomas A. Delomas, John H. Powell, Jon E. Hess, Shawn R. Narum, Matthew R. Campbell

**Affiliations:** ^1^ Pacific States Marine Fisheries Commission Eagle Idaho USA; ^2^ U.S. Department of Agriculture Agricultural Research Service National Cold Water Marine Aquaculture Center Kingston Rhode Island USA; ^3^ Idaho Department of Fish and Game Eagle Idaho USA; ^4^ Columbia River Inter‐Tribal Fish Commission Portland Oregon USA; ^5^ Columbia River Inter‐Tribal Fish Commission Hagerman Idaho USA

**Keywords:** endangered species act, management, microhaplotypes, *Oncorhynchus mykiss*, single‐nucleotide polymorphisms, Snake River basin, steelhead

## Abstract

Genetic stock identification (GSI) is an important fisheries management tool to identify the origin of fish harvested in mixed stock fisheries. Periodic updates of genetic baselines can improve performance via the addition of unsampled or under‐sampled populations and the inclusion of more informative markers. We used a combination of baselines to evaluate how population representation, marker number, and marker type affected the performance and accuracy of genetic stock assignments (self‐assignment, bias, and holdout group tests) for steelhead (*Oncorhynchus mykiss*) in the Snake River basin. First, we compared the performance of an existing genetic baseline with a newly developed one which had a reduced number of individuals from more populations using the same set of markers. Self‐assignment rates were significantly higher (*p* < 0.001; +5.4%) for the older, larger baseline, bias did not differ significantly between the two, but there was a significant improvement in performance for the new baseline in holdout results (*p* < 0.001; mean increase of 25.0%). Second, we compared the performance of the new baseline with increased numbers of genetic markers (~2x increase of single‐nucleotide polymorphisms; SNPs) for the same set of baseline individuals. In this comparison, results produced significantly higher rates of self‐assignment (*p* < 0.001; +9.7%) but neither bias nor leave‐one‐out were significantly affected. Third, we compared 334 SNPs versus opportunistically discovered microhaplotypes from the same amplicons for the new baseline, and showed the latter produced significantly higher rates of self‐assignment (*p* < 0.01; +2.6%), similar bias, but slightly lower holdout performance (−0.1%). Combined, we show the performance of genetic baselines can be improved via representative and efficient sampling, that increased marker number consistently improved performance over the original baseline, and that opportunistic discovery of microhaplotypes can lead to small improvements in GSI performance.

## 1 INTRODUCTION

For fish species subject to mixed stock fisheries, genetic stock identification (GSI) is a widely applied tool to infer the proportion of individuals originating from potential source populations. The application of GSI involves genotyping samples from potential sources which are grouped into genetic stocks, or reporting units, and samples of unknown origin (e.g., a mixed stock fishery) are subsequently genotyped and compared against the reference baseline (Anderson et al., 2008; Manel et al., 2005). The application of GSI in marine and freshwater environments is commonplace (Beacham et al., 2008; Campbell et al., 2012; Ruzzante et al., 2000) and provides critical information to fisheries managers about the relative abundance of stocks and their associated levels of harvest (e.g., Beacham et al., 2017, 2019; Hasselman et al., 2016). Given the central role of GSI in managing many fisheries, the accuracy and reliability of these methods are important.

Empirical and simulation studies have identified several factors that can influence the accuracy of GSI assignments. Examples include the number of sampled individuals and populations (Beacham et al., 2011), levels of genetic differentiation among reference populations (Araujo et al., 2014), and the numbers and types of molecular markers used in analysis (Ackerman et al., 2011; Larson et al., 2014; McKinney et al., 2017, 2020). The numbers of samples per population can impact estimates of population‐level allele frequencies (Beacham et al., 2011; Kalinowski, 2004; Winans et al., 2004) and different numbers of samples are needed to obtain equivalent levels of accuracy for different marker types (e.g., microsatellites vs single‐nucleotide polymorphisms). Further, within a given marker type, it is possible that selective forces may render some markers more informative than others. For example, single‐nucleotide polymorphisms (SNPs) subject to diversifying selection in Sockeye salmon (*Oncorhynchus nerka*) increased assignment resolution relative to a panel of neutral SNP markers (Ackerman et al., 2011). Additionally, levels of genetic differentiation (e.g., *F*_ST_) have been shown to affect the accuracy of genetic stock identification, with low levels of genetic differentiation between populations (e.g., *F*_ST_ <0.01) leading to significant bias (>15%) in stock assignments (Araujo et al., 2014). Taken together, a number of factors can influence GSI assignment accuracy and warrant consideration when constructing new baselines or updating existing ones (e.g., Anderson et al., 2008).

For long‐term genetic monitoring projects, genetic baselines are periodically revised to update allele frequencies, add unsampled (or under‐sampled) populations, or add additional markers. Because molecular marker development is an ongoing process, baseline updates also represent an opportunity to add novel markers that capture additional variation (e.g., Collins et al., 2020) to increase the accuracy of genetic stock assignments (McKinney et al., 2017). Updating baselines often represents a significant expenditure, and so it is important to consider approaches that will maintain or increase GSI accuracy while minimizing costs.

One method of controlling costs is to utilize efficient genotyping approaches, such as amplicon sequencing (e.g., GT‐seq; Campbell et al., 2015). This technique makes it possible to sequence hundreds of SNPs for thousands of individuals at drastically reduced costs (≈ $4 USD per sample) relative to previous technologies (e.g., 5′ exonuclease assays; Campbell et al., 2019). In addition, if amplicons contain two or more SNPs, bioinformatic pipelines that analyze sequencing data can genotype these in a phase‐aware manner to yield haplotypes (Leitwein et al., 2020). The utilization of multi‐SNP haplotypes covering a small genomic region (i.e., a single amplicon), also known as microhaplotypes (Baetscher et al., 2018), is a significant advance as they increase the number of independent alleles in an analysis which can increase precision in estimates of genetic distance (Kalinowski, 2002) and provide greater statistical power than traditional bi‐allelic SNPs (Oldoni et al., 2019). Therefore, using amplicon sequencing to build a genetic baseline allows any amplicons containing multiple SNPs to be opportunistically genotyped as microhaplotypes with no additional cost.

Microhaplotypes have shown promise in several fisheries‐related studies including pedigree inference and population assignment. Inclusion of microhaplotype data can increase power for identifying kin relationships (Baetscher et al., 2018; Delomas & Campbell, 2022; Euclide et al., 2022) and fewer microhaplotypes are necessary to obtain equivalent levels of accuracy with parentage analysis relative to bi‐allelic SNPs (May et al., 2020). Similarly, added variability associated with microhaplotypes has the potential to differentiate closely‐related populations, resulting in increased accuracy in GSI assignments (Euclide et al., 2021; McKinney et al., 2020). Lastly, the inclusion of more genetic markers, as opposed to additional individuals, has been shown to be more impactful in reducing bias in GSI assignments (Landguth et al., 2012; Powell et al., 2018). Combined, this suggests baselines could be improved by including more markers displaying greater variability while controlling for costs by genotyping a reduced number of samples. Changes to sample sizes could occur by altering the number of individuals per collection or the number of collections per reporting unit, both of which could alter the representation of different management populations in the GSI baseline.

The Snake River basin encompasses portions of Idaho, Oregon, and Washington, and once produced more than half of all natural‐origin steelhead returning to the Columbia River basin (CRB) which exceeded 500,000 fish annually (Chapman, 1986; Mallet, 1974). Dramatic declines in abundance prompted the listing of the natural‐origin Snake River basin steelhead distinct population segment (DPS) as threatened in 1997 (62 FR 43937). Despite significant declines, the Snake River basin still produces a substantial portion of the steelhead in the CRB, comprising an average of 50.2% of summer steelhead that pass the first dam on the Columbia River (Bonneville Dam) and the last dam on the lower Snake River between 2001–2020 (Lower Granite Dame; ODFW and WDFW, 2022). As part of ongoing recovery efforts, genetic monitoring plays a critical role in the management of steelhead in both the Snake and Columbia River basins. For example, GSI and parentage‐based tagging (PBT) have become instrumental in characterizing the abundance, distribution, and life‐history characteristics of both natural‐ and hatchery‐origin steelhead (Campbell et al., 2012; Copeland et al., 2017; Coykendall et al., 2022; Hargrove, Camacho, et al., 2021; Steele et al., 2013, 2019).

When steelhead populations in the Snake River basin were first described, genetic data were limited and populations were developed based on available genetic data, management applications, and geographic features (ICTRT, 2003). Since that time the genetic structure of steelhead in the Snake River basin has been characterized using microsatellite (Blankenship et al., 2011; Campbell et al., 2012) and SNP marker panels of varying size (Matala et al., 2014; Powell et al., 2018 and references therein). Overall, steelhead in the Snake River basin display low levels of genetic differentiation (mean pairwise *F*_ST_ across populations = 0.025; Hargrove, Delomas, et al., 2021) and populations occupying terminal (uppermost) portions of river drainages exhibit higher levels of genetic differentiation relative to downstream portions (e.g., Campbell et al., 2012; Hargrove, Delomas, et al., 2021; Nielsen et al., 2009). Additionally, the genetic structure of select basins has been altered by hatchery translocations and/or supplementation (e.g., upper Salmon River; Powell & Campbell, 2020). Currently, multiple state, federal, and tribal agencies collaboratively genotype steelhead from the Snake and Columbia River basins as part of annual monitoring efforts, and a standard marker panel has been adopted for use throughout the system (Hess et al., 2023). Recently, an expanded bi‐allelic SNP marker panel was developed for steelhead, and concomitantly, efforts were initiated to update the GSI baseline for steelhead in the Snake River basin using this expanded marker panel.

In this study, we examined the influence of population representation (number of populations and individuals per population), marker number, and marker type on GSI performance using a case study of steelhead in the Snake River basin. Steelhead represent an interesting study system in that individual distinct population segments are distributed across broad spatial scales, follow an isolation by distance model of genetic structure, and have been subject to widespread hatchery augmentation efforts. These characteristics imply GSI performance could vary by stock as a result of natural and anthropogenic gene flow, a pattern common to many fish species. To address our research questions, we compared the efficacy of two distinct genetic baselines, both of which represent the same reporting units, but which contained different numbers of populations and number of individuals per population. The previous genetic baseline contained 5967 individuals that were grouped into 45 populations based on genetic similarity. In contrast, the new baseline consisted of almost half as many individuals (3150 individuals, 44 populations) but covered a broader geographic area within the basin. Both baselines were genotyped at a common set of 176 SNPs, but an additional 158 SNPs were available for the new baseline. We assessed the impact of population representation through comparisons of the two baselines at the same marker panel. Next, we tested the impact of marker number on GSI by comparing performance of the new baseline at 176 and 334 SNPs. Lastly, we examined the role of marker type by calling a subset of bi‐allelic SNPs (*n* = 91) from the new marker panel (*n* = 334) as either conventional SNPs or microhaplotypes (i.e., 334 SNPs vs. 243 SNPs plus 91 microhaplotypes) for the new baseline. For each baseline‐marker combination we used self‐assignment tests to determine the proportion of samples that were assigned to their known reporting unit of origin, performed simulations to quantify assignment bias, and used hold‐out data sets (collections of returning adults with known spawning locations) to estimate concordance between GSI assignments and last known location. Our expectation was that the new baseline with microhaplotypes would generate the highest self‐assignment rates and lowest bias. Because of the broader population coverage in the new baseline, we expected it to yield higher concordance rates between last known location and GSI assignments compared to the older baseline.

## 2 METHODS

### 2.1 GSI of Snake River Basin steelhead

**FIGURE 1:**
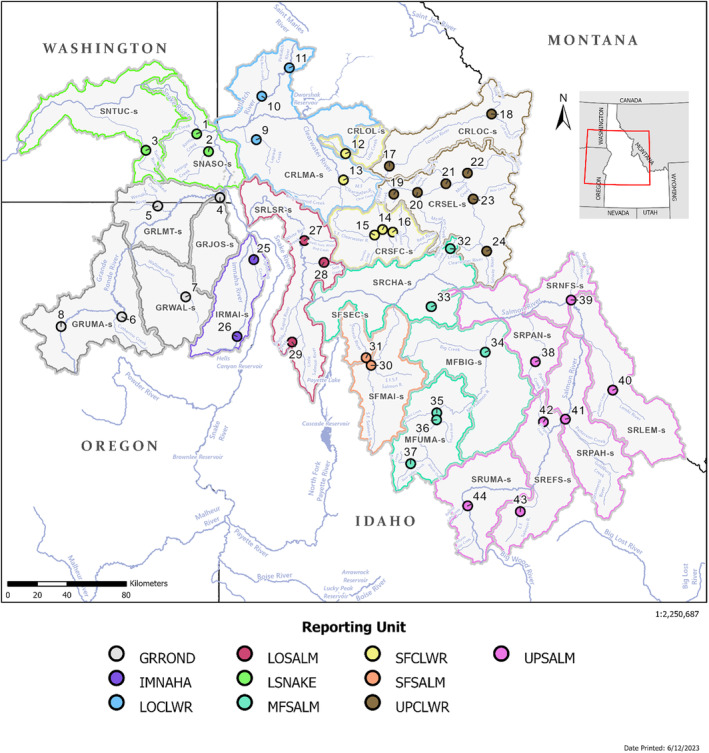
A map of the 24 extant Snake River basin steelhead (*Oncorhynchus mykiss*) management populations (outlined in polygons) and 10 genetic reporting units (polygon color) used for genetic stock identification. Details associated with population codes (abbreviations ending in ‘‐s’) and reporting unit names can be found in Table 1, and numbers correspond to collections of individuals included in genetic baseline v4. The previous genetic baseline (version 3.1) lacked representation for the upper Grande Ronde River population (GRUMA‐s, collection numbers 6, 8) and the Panther Creek population (SRPAN‐s, collection number 40).

The Snake River Basin steelhead DPS was listed as threatened by the National Marine Fisheries Service (NMFS)/National Oceanic and Atmospheric Administration (NOAA) in 1997 (62 FR 43937), and associated with these efforts, the Interior River Columbia Technical Recovery Team (ICTRT) identified management populations which were used to establish criteria to assess viability (ICTRT, 2003). There are 24 extant management populations of steelhead in the Snake River basin distributed across the Snake, Grande Ronde, Imnaha, Clearwater, Salmon Rivers, and their associated tributaries (Figure 1; Table 1).

**TABLE 1:** Details associated with number of collections and individuals included in the Snake River basin steelhead (*Oncorhynchus mykiss*) genetic baselines v3.1 and v4 organized by reporting unit and management population.

Reporting unit	Reporting unit code	Management population	Population code	Number of genetic populations		Number of individuals	
				v3.1	v4	v3.1	v4
lower Snake River	LSNAKE	Tucannon River	SNTUC‐s	2	1	204	89
		Asotin Creek	SNASO‐s	1	2	194	163
Grande Ronde River	GRROND	lower Grande Ronde	GRLMT‐s	2	1	264	77
		Joseph Creek	GRJOS‐s	1	1	98	100
		upper Grande Ronde	GRUMA‐s*	0	2	0	321
		Wallowa River	GRWAL‐s	3	1	322	74
Imnaha River	IMNAHA	Imnaha River	IRMAI‐s	3	2	276	128
lower Clearwater River	LOCLWR	lower main Clearwater River	CRLMA‐s	5	4	695	308
South Fork Clearwater River	SFCLWR	Lolo Creek	CRLOL‐s	1	1	94	46
		South Fork Clearwater River	CRSFC‐s	3	3	282	155
upper Clearwater River	UPCLWR	Lochsa River	CRLOC‐s	2	2	276	91
		Selway River	CRSEL‐s	4	6	666	341
lower Salmon River	LOSALM	Little Salmon and Rapid River	SRLSR‐s	2	3	222	116
South Fork Salmon River	SFSALM	South Fork Salmon River	SFMAI‐s	2	1	181	47
		Secesh River	SFSEC‐s	1	1	208	63
Middle Fork Salmon River	MFSALM	lower Middle Fork Salmon River	MFBIG‐s	2	1	321	47
		upper Middle Fork Salmon River	MFUMA‐s	4	3	726	149
		Chamberlain Creek	SRCHA‐s	1	2	189	156
upper Salmon River	UPSALM	East Fork Salmon River	SREFS‐s	2	2	146	109
		Lemhi River	SRLEM‐s	1	1	86	53
		North Fork Salmon River	SRNFS‐s	1	1	100	100
		Pahsimeroi River	SRPAH‐s	1	1	98	316
		Panther Creek	SRPAN‐s*	0	1	0	50
		Upper Mainstem Salmon River	SRUMA‐s	1	1	319	51
Totals				45	44	5937	3150

*Note*: Management population refers to the 24 extant steelhead populations recognized by the Interior River Columbia Technical Recovery Team (ICTRT). Genetic populations correspond to groups of collections whose level of genetic differentiation were not significantly different. Populations with an asterisk were those added to GSI baseline v4 but were missing from baseline v3.1.

Historical efforts to monitor returns of adult steelhead to the Snake River basin using counting methodologies (e.g., weirs and redd count surveys) have been limited by environmental conditions during the spawning season (e.g., high turbidity and changing flow conditions; Thurow, 1985). In response, genetic stock identification has become a critical tool for estimating adult escapement and was first implemented by Idaho Department of Fish and Game in the Snake River basin in 2009 (Ackerman & Campbell, 2010). Since then, four different genetic baselines have been developed, which have differed in management population representation, the number of samples analyzed, and the number of genetic markers assayed.

The Snake River basin GSI baseline version 3.1 was developed in 2014 and contained 5967 individuals from 136 collection events (Powell et al., 2018; Vu et al., 2015). As is common in GSI analysis, collections of individuals were grouped into populations based on patterns of genetic differentiation and management objectives which were then organized into reporting units or genetic stocks (e.g., Seeb et al., 2007). For the Snake River basin GSI baseline v3.1, individuals were partitioned into 45 populations and 10 reporting units: (1) lower Snake River, (2) Grande Ronde River, (3) Imnaha River, (4) lower Clearwater River, (5) South Fork Clearwater River, (6) upper Clearwater River, (7) lower Salmon River, (8) Middle Fork Salmon River, (9) South Fork Salmon River, and (10) upper Salmon River. This baseline represented 22 of the 24 management populations in the basin but lacked samples from Panther Creek or the upper Grande Ronde River (Table 1). The Snake River basin GSI baseline v3.1 and associated metadata are available at fishgen.net (McCane et al., 2018; fishgen ID: “Snake River steelhead GSI baseline version 3.1”) including details associated with the 179 SNP marker panel at which samples were amplified (fishgen ID: “CRITFC/IDFG steelhead 192 GSI v4.1 + PBT v5.1”).

Recently, a new GSI baseline (version 4) was developed for steelhead which consisted of 3150 samples from 76 collection events which were placed into 44 genetic populations. In contrast to baseline v3.1, all 24 management populations in the Snake River basin were represented (Table 1). The same set of reporting units were used for baselines v3.1 and v4. Baseline version 4 was characterized at a set of 334 SNP loci, a subset of which (91) were called as microhaplotypes (see “*Genotyping and microhaplotype discovery*” section below). A detailed description of this baseline (i.e., levels of diversity, genetic differentiation, etc.) can be found in Hargrove, Delomas, et al. (2021), with the only modification being the removal of two collections (Big Bear Creek and West Fork Potlatch River). Big Bear Creek was dropped due to low sample sizes (*n* = 9) and individuals from West Fork Potlatch River were dropped as they were genetically similar (*F*_ST_ = 0.002) to the East Fork Potlatch River and their removal did not impact GSI performance. A copy of the Snake River basin GSI baseline v4 has been provided in File S1. All samples were amplified at a panel of 368 bi‐allelic SNPs, the details of which (locus names, probe sequence files, and SNP positions) can be downloaded at fishgen.net (fishgen ID: ‘CRITFC IDFG steelhead GT‐seq v5.0 368’). As noted above, the final version of baseline v4 included 334 loci, 91 of which were called as microhaplotypes. A list of markers used in baseline v4 and their characteristics (e.g., marker type, heterozygosity, and fixation index) can be found in File S2. A copy of microhaplotype details (SNP positions and probe sequence files necessary for calling microhaplotypes) has been provided in Files S3 and S4.

Marker panels used for monitoring steelhead in the Snake River basin have been periodically expanded but new panels remain back compatible with previous ones. In other words, the SNPs amplified in baseline v3.1 were included in the marker panel used for baseline v4. In rare instances, interactions between new and old loci during PCR amplification result in old loci being dropped from the updated panel due to high failure rates. Three SNPs were dropped from the 179 SNP panel when our marker panel was expanded, and therefore GSI analyses were performed on a panel of 176 SNPs (dropped loci: Omy_aldB165, Omy_colla1525, Omy_CRBF11).

### 2.2 Genotyping and microhaplotype discovery

Genomic DNA was extracted from fin clips using the nexttec Genomic DNA Isolation Kit (XpressBio, Thurmont, Maryland) following the manufacturer's protocol. Samples were amplified at a panel of 368 single‐nucleotide polymorphisms (SNPs) (fishgen ID: ‘CRITFC IDFG steelhead GT‐seq v5.0 368’) using the Genotyping‐in‐Thousands by sequencing (GT‐seq) protocol described in Campbell et al. (2015). Library preparation began with an initial multiplex PCR reaction to ligate a pair of sequencing primers to the target sequences which contain a known single‐nucleotide polymorphism (SNP). In a subsequent PCR reaction, the sample was “barcoded” by ligating an additional sequence to the target that identifies the sample's tray of origin (i7 barcode) and its position on the tray (i5 barcode). After barcoding, the SequalPrep™ Normalization Plate Kit (Applied Biosystems) was used to bind a standard amount of amplicon product and normalize concentrations. A total of 96 samples were pooled into each single “plate library.” All plate libraries were quantified by a Qubit fluorometer (Invitrogen), and concentrations were normalized again before being pooled. Loci were genotyped by sequencing the target location on the Illumina NextSeq. A bioinformatics pipeline was used to assign resulting sequences and the genotypes back to individual samples using the unique combination of i5 and i7 barcodes.

Because the GT‐seq approach employs amplicon sequencing which produces short reads (~150 base pairs) surrounding a SNP of interest, this affords the opportunity to look for additional SNPs linked to a primary SNP of interest (i.e., a microhaplotype). Reference amplicon sequences were created by pooling reads for nine samples and, for each locus, extracting the unique sequence with highest depth that began with the forward primer and contained one of the in silico probes. Reads for all samples were aligned to these reference sequences with bowtie2 (Langmead & Salzberg, 2012) using the following parameters: ‐‐end‐to‐end ‐N 1 ‐‐rdg 0,5 ‐‐rfg 0,5 ‐‐score‐min L,0,‐0.76. The main function of these parameters compared to the defaults was to allow alignments to be made with more differences between the read and the reference. This was justified as the amplicons were expected to contain one or more polymorphisms and the relatively small number of amplicon sequences implied that reads originating from different regions were not likely to be highly similar by chance. Only reads matching the forward strand were retained.

In addition to the known SNPs in these loci, candidate substitution SNPs were identified by utilizing the “mpileup” and “call” routines in samtools/bcftools 1.9 (Li, 2011). Loci with more than 8 candidate SNPs were removed to exclude primer sets that were potentially amplifying multiple paralogous sequences. Individuals were then genotyped for the candidate SNPs using a “microhaplotype aware” amplicon sequencing genotyper, microTyper (https://github.com/delomast/microTyper). Posterior probabilities for each genotype were calculated using a uniform prior, a multinomial likelihood with errors considered equally likely to be reads of any other allele and assuming an error rate of 0.01 (1%). Genotypes were called when the posterior probability was greater than 0.99 and depth was greater than or equal to 10 reads.

Multiple approaches were used to exclude loci from consideration as microhaplotypes if they were potentially amplifying multiple paralogous sequences. First, read count plots were visually evaluated and loci were removed if they showed either a systematic deviation of allele balance in heterozygotes from 0.5 or distinct clusters of heterozygotes with different mean allele balances. Then, non‐variable candidate SNPs (as genotyped by microTyper) were removed. Finally, deviation from Hardy–Weinberg equilibrium was assessed with permutation tests (Graffelman & Weir, 2018) within each collection; any locus with a significant deviation from HWE (FDR < 0.05, Benjamini & Yekutieli, 2001) in two or more collections were removed.

### 2.3 Baseline and marker panel comparisons

Our primary objective was to evaluate the influence of population representation, marker number, and marker type on the performance of GSI in the Snake River basin. To this end we performed GSI using two separate genetic baselines and three different combinations of markers (Table 2). As noted above, different collections of individuals were used for the two baselines, but the hierarchical structure of collections and their placement into genetic reporting units based on geographic location was consistent across baselines.

**TABLE 2:** Combinations of marker panels and genetic baselines used to assess the impact of population representation, marker number, and marker type on GSI performance.

Variable tested	Baseline‐marker combination				Performance metric					
	Baseline	Samples	Populations	Markers	Self‐assignment	*p*‐value	Bias	*p*‐value	Concordance	*p*‐value
Population representation	3	5962	22	176 SNP	77.4	<0.001	1.68 × 10^−18^	0.63	62.6	< 0.001
	4	3150	24	176 SNP	72.0		1.52 × 10^−19^		87.6	
Marker number	4	3150	24	176 SNP	72.0	< 0.001	1.52 × 10^−19^	0.89	87.6	0.07
	4	3150	24	334 SNP	81.7		−2.35 × 10^−18^		85.6	
Marker type	4	3150	24	334 SNP	81.7	< 0.01	−2.35 × 10^−18^	<0.01	85.6	1.00
	4	3150	24	334 SNP/microhap	84.3		6.04 × 10^−18^		85.5	

*Note*: Shaded boxes highlight the variables which differ between baseline‐marker comparisons. *p*‐values correspond to output from pairwise tests of significance (see methods for specific tests used for individual comparisons).

To evaluate the impacts of marker panel on GSI, we first performed GSI related analyses using the Snake River basin genetic baseline v3.1 (Hargrove, Delomas, et al., 2021; Powell et al., 2018; Vu et al., 2015) which contained 176 SNPs that were common to both baselines (hereafter: v3‐176 SNP). Next, we performed genetic stock identification using baseline v4 at three different sets of markers; (1) 176 SNPs (hereafter: v4‐176 SNP; File S5), (2) 334 bi‐allelic SNPs (hereafter: v4‐334 SNP; File S6), and (3) 91 microhaplotypes and 243 SNPs (hereafter: v4‐334 SNP/microhaplotype). For both baseline v3.1 and v4, all samples used in analyses were amplified at >90% of loci in their respective SNP panel and were not genetic duplicates of another sample within that baseline.

To determine the ability of different marker panels to differentiate between reporting units we estimated *F*_ST_ using the algorithms of Weir and Cockerham (1984) as implemented in the R package *hierfstat* (function “wc”) on a per locus basis.

### 2.4 Self‐assignment rates

We performed self‐assignment tests using a leave‐one‐out procedure as implemented in the “self_assign” function in the R package *rubias* (Anderson et al., 2008; Moran & Anderson, 2019) to determine the rate at which individuals were correctly assigned to their reporting unit of origin. In self‐assignment tests, individuals from the baseline are removed one at a time, baseline allele frequencies are re‐calculated with that individual removed, and the population (and reporting unit) of origin of that individual is then estimated. Self‐assignment tests were performed on four different baseline and marker combinations which included, (1) v3‐176 SNP, (2) v4‐176 SNP, (3) v4‐334 SNP, and (4) v4‐334 SNP/microhaplotype. Individuals were assigned to the reporting unit with the highest probability regardless of assignment probability (i.e., we employed no minimum threshold). To assess the relative impact of population representation, marker number, and marker type on self‐assignment rates, we coded assignments as either correct or incorrect based on whether an individual fish was assigned to its correct reporting unit of origin. We then performed three pairwise tests using the four baseline/marker combinations using a Chi‐square test in R (R Core Team, 2021). The paired tests were as follows: (1) v3‐176 SNP versus v4‐176 SNP (population representation); (2) v4‐176 SNP versus v4‐334 SNP (marker number); and (3) v4‐334 SNP versus v4‐334 SNP/microhaplotype (marker type).

### 2.5 Simulated mixtures and GSI accuracy

In addition to self‐assignment tests, we characterized the accuracy of GSI assignments via simulation of fishery mixtures using baseline allele frequencies following the leave‐one‐out approach of Anderson et al. (2008). We simulated 1000 mixtures equal to 1000 individuals using the “assess_reference_loo()” function in *rubias* with flat priors (Dirichlet distribution, parameters = 1.5) to determine the proportion of samples drawn from different reporting units. For each comparison, error was estimated as the difference between the true simulated mixture proportion from each reporting unit (expected) and the estimated proportion of samples assigned to each reporting unit (observed). We performed the same set of pairwise tests to determine if accuracy was significantly different between marker/baseline combination using paired Mann–Whitney U tests in R.

### 2.6 Concordance between passive integrated transponder (PIT) tagged fish and GSI assignments

Lastly, we used a collection of returning adult steelhead for which both genetic samples and PIT tag location data were available to assess what fraction of individuals that were last detected at a specific location on the landscape via PIT tag were also assigned to the genetic reporting unit associated with that location via GSI. Samples in this hold‐out training set were independent of steelhead used in the construction of our GSI baseline. A concordance rate was estimated as the proportion of fish last detected in each reporting unit via PIT tag which were also assigned to the same reporting unit via GSI. We assumed concordance between last known PIT tag location and GSI assignments could be used as a proxy for accuracy of a GSI baseline because natural‐origin steelhead that enter the Snake River and pass Lower Granite Dam exhibit low rates of straying from natal streams (Keefer & Caudill, 2012).

Details associated with sampling of returning adults and PIT tag detection on the landscape are as follows. A subset of all adult steelhead returning to the Snake River basin were sampled for biological data and genetics at Lower Granite Dam as part of a multi‐agency monitoring project to track stock abundances (see Hargrove, Delomas, et al., 2021 and references therein). As part of the routine biological sampling, fish are diverted into an adult fish trap as they make their way up the fish passage ladder. Once in the adult fish trap, a fin clip (for genotyping) was taken, and a PIT tag implanted into each adipose intact (putatively natural‐origin) adult. After sampling, adult steelhead were returned to the fish ladder where they continued their upstream spawning migration. Fish were later detected at PIT tag detection arrays distributed throughout the Snake River basin in various rivers and streams and the physical location of each PIT tag array was related to the boundaries of management populations. As noted above, a GSI assignment was concordant with a PIT tag detection if they both corresponded to the same GSI reporting unit. Concordance was estimated using returning adults sampled during spawn year 2019 and 2020.

Each PIT‐tagged individual was genotyped at the same 368 SNP marker panel using the same protocols as for the GSI baseline. As with baseline samples, duplicate individuals were removed, and each sample needed to be genotyped at a minimum of 90% of loci to be retained. Fish were assigned to one of 10 reporting units via individual assignment procedures using the “infer_mixture” function implemented in the R package *rubias* (Moran & Anderson, 2019) with 4000 reps following 2000 burn in iterations. Individuals were assigned to the reporting unit with the highest assignment probability regardless of its value.

We compiled a list of last known detection site for PIT‐tagged individuals from spawn year 2019 and 2020, and these locations were assumed to represent their management population of natal origin. Paired GSI and PIT tag detection data were available for a total of 1981 returning steelhead adults from spawn years 2019 (894) and 2020 (1087). The physical location for PIT tag detection arrays were related to genetic reporting units based on their geographic locations. We elected to drop PIT tag observations from the Lower Salmon reporting unit as detections were too few (n = 2) for meaningful inference. As with previous analyses, we performed these analyses using the four different combinations of marker panels and genetic baselines described above (v3‐176 SNP, v4‐176 SNP, v4‐334 SNP, and v4‐334 SNP/microhaplotype). We coded fish as ‘concordant’ or ‘discordant’ based on whether the fish was assigned to the same reporting unit based on PIT tag and GSI assignments. We tested for differences between baselines combinations via Chi‐square tests in R.

## 3 RESULTS

The Snake River basin steelhead baseline (v3.1) had an average of 133 individuals (range: 39–319) per population and 597 individuals (range: 222–1236) per reporting unit (Table 1). When aggregated into management populations, this baseline had an average of 271 individuals (range: 86–726) for 22 of the 24 extant management populations.

The Snake River basin steelhead baseline v4 consisted of 3150 with an average number of 72 individuals (range: 23–316) per population and 315 individuals (range: 110–680) per reporting unit (NMFS, 2017; Table 1; Figure 1). On a population management basis, all 24 populations were represented with an average of 131 individuals (range: 46–341).

The number of loci in the updated marker panel which exhibited three or more alleles was 91. Per locus estimates of *F*_ST_ were right skewed regardless of panel but were highest for v4‐334 SNP (avg *F*_ST_ = 0.0354) which was minimally different than v4‐SNP/microhaplotype (avg *F*_ST_ = 0.0353). Estimates of *F*_ST_ were lower for both v3‐176 SNP and v4‐176 SNP (Table 3).

**TABLE 3:** Average estimates of *F*_ST_ per locus summarized as a function of GSI baseline version and marker panel for steelhead (*Oncorhynchus mykiss*) in the Snake River basin.

Baseline	Marker panel	Average	Range	Stand. Dev.
3	176 SNP	0.0335	0.0073 to 0.1039	0.0181
4	176 SNP	0.0315	0.0008 to 0.1020	0.0158
4	334 SNP	0.0354	−0.0012 to 0.3503	0.0316
4	334 SNP/MH	0.0353	−0.0012 to 0.3368	0.0298

Abbreviations: MH, microhaplotype; SNP, single‐nucleotide polymorphism.

### 3.1 Self‐assignment rates

**FIGURE 2:**
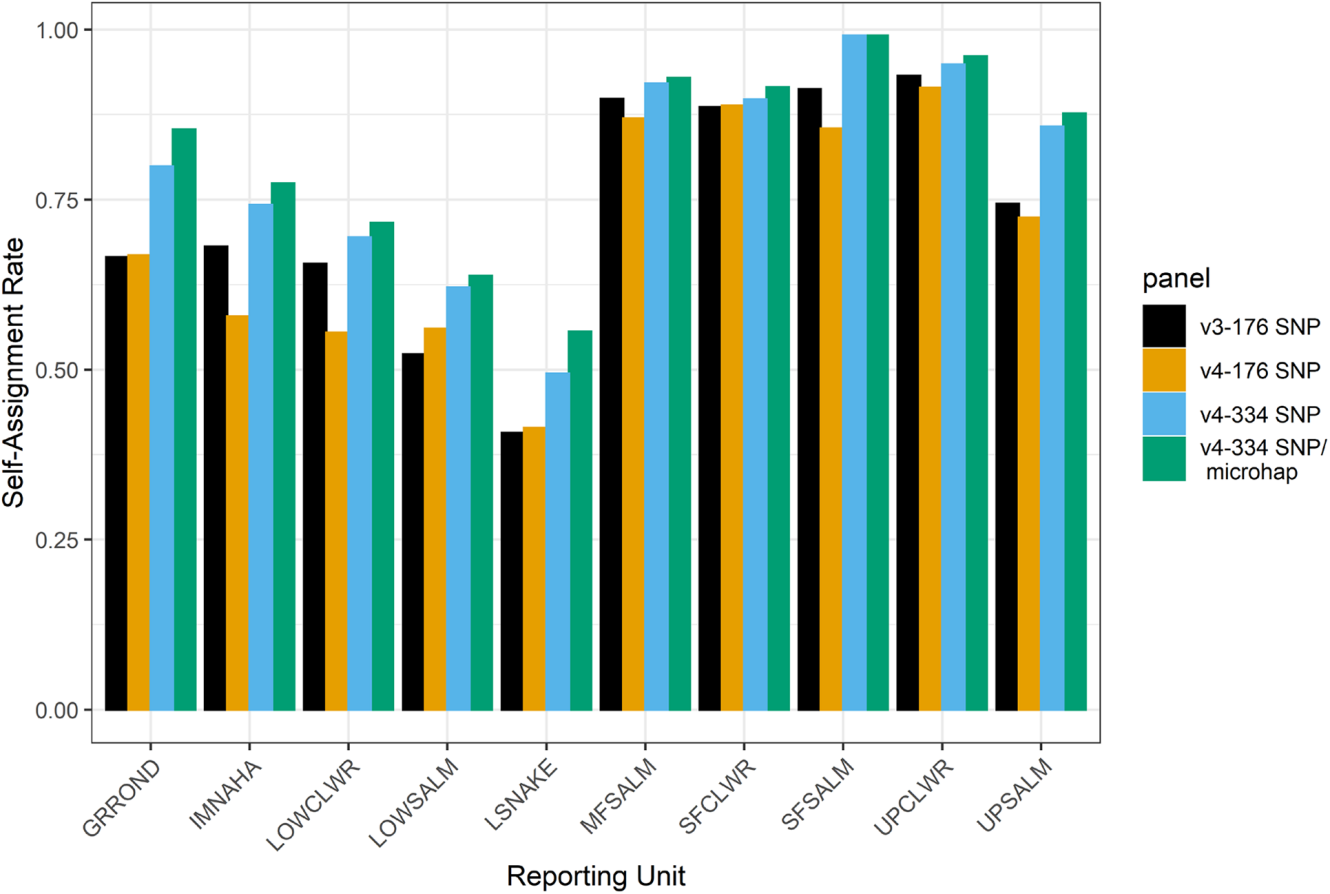
Self‐assignment rates for steelhead (*Oncorhynchus mykiss*) from the Snake River basin as a function of genetic baselines (versions 3.1 and 4), marker numbers (176 versus 334 SNPs), and marker types (334 SNPs versus 243 SNPs and 91 microhaplotypes). Abbreviations for reporting units are as follows: GRROND = Grande Ronde River, IMNAHA = Imnaha River, LOWCLWR = lower Clearwater River, LOWSALM = lower Salmon River, LSNAKE = lower Snake River, MFSALM = Middle Fork Salmon River, SFCLWR = South Fork Clearwater River, SFSALM = South Fork Salmon River, UPCLWR = Upper Clearwater River, and UPSALM = Upper Salmon River.

The number of fish that correctly assigned to their reporting unit of origin was significantly higher for the older, larger baseline (v3‐176 SNP; 4620 fish or 77.4% of baseline) relative to the newer, smaller baseline (v4‐176 SNP; 2268 fish or 72.0% of baseline; Table 2, Figure 2) at the α = 0.05 level (χ^2^ = 32.29, df = 1, *p*‐value <0.001). Differences in self‐assignment rates were also significantly higher when more SNPs (334 vs. 176) were analyzed for the newer baseline (χ^2^ = 81.94, df = 1, *p*‐value <0.001). Specifically, the percentage of fish correctly assigned to the larger marker panel (v4‐334 SNPs) was 81.7% relative to a panel of 176 SNPs (72.0%). Lastly, self‐assignment rates were significantly higher (χ^2^ = 7.63, df = 1, *p*‐value <0.01) when microhaplotypes were included (v4‐334 SNP/microhaplotypes; correct assignments = 84.3%) compared to a panel of bi‐allelic SNPs (v4‐334 SNPs). Changes in self‐assignment rates as a function of baseline‐marker‐marker type combination differed across reporting units (Figure 2). When comparing results from the old baseline relative to different iterations of the newly developed one, the least amount of improvement in self‐assignment rates observed in the South Fork Clearwater River, upper Clearwater River, and Middle Fork Salmon River. In contrast, self‐assignment rates in the Grande Ronde River, lower Snake River, and upper Salmon River were much higher for the new baseline relative to the previous iteration.

### 3.2 Simulated mixtures and GSI accuracy

**FIGURE 3:**
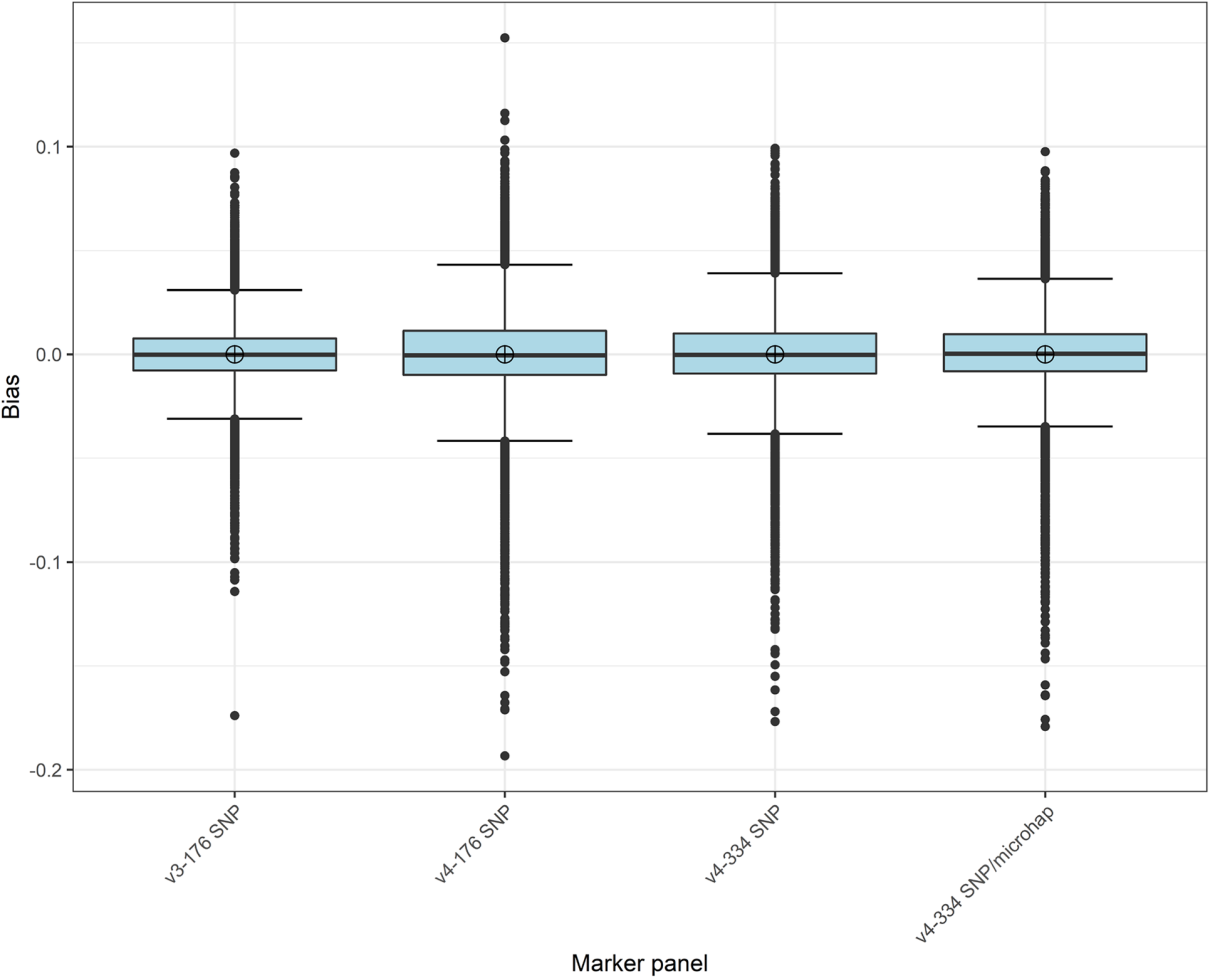
Bias associated with genetic stock identification assignments made based on simulated mixtures of steelhead (*Oncorhynchus mykiss*) for different marker panels. Open circles denote mean values, horizontal bars denote median values, lower and upper hinges correspond to the first and third quartiles, and whiskers represent values 1.5 times the inter‐quartile range.

**FIGURE 4:**
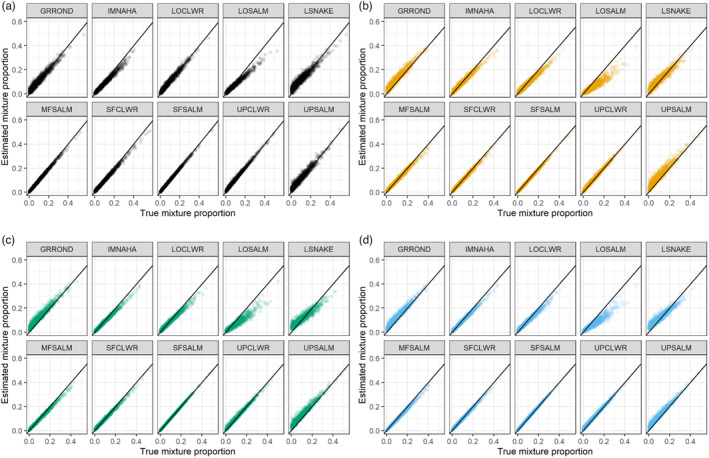
A comparison of true mixture proportions and simulated mixture proportions by reporting unit for four separate baseline and marker combinations, a) baseline v3‐176 SNPs, b) baseline v4‐176 SNPs, c) baseline v4‐334 SNP, d) baseline v4‐334 SNP/microhaplotypes. For a description of reporting unit abbreviations, see Figure 2.

The accuracy of GSI assignments was high regardless of marker panel (Table 2, Figure 3), with only select reporting units displaying evidence of overestimation (Grande Ronde, Upper Salmon, and Lower Snake rivers) or underestimation (Lower Salmon River) depending on known mixture proportions (Figure 4). Bias was not significantly different (W = 49,536,294, *p*‐value = 0.26) at the α = 0.05 level when comparing the older, larger baseline (v3‐176 SNP, average bias = 1.46 × 10^−19^, SD = 0.02) with the new baseline typed at the same marker panel (v4‐176 SNP, average bias = 4.02 × 10^−18^, SD = 0.03). For the newer baseline, accuracy of GSI assignments was not significantly different (W = 50,139,467, *p*‐value = 0.73) for the larger marker panel (v4‐334 SNP; average bias = 1.51 × 10^−18^, SD = 0.02) relative to the smaller marker panel (v4‐176 SNP). Lastly, bias was not significantly different (W = 49,346,238, *p*‐value = 0.11) for the new baseline with a combination of SNPs and microhaplotypes (v4‐334 SNP/microhaplotypes; average bias = 9.90 × 10^−18^, SD = 0.02) relative to the same number of bi‐allelic SNPs (v4‐334 SNP).

### 3.3 GSI and PIT tag concordance

**FIGURE 5:**
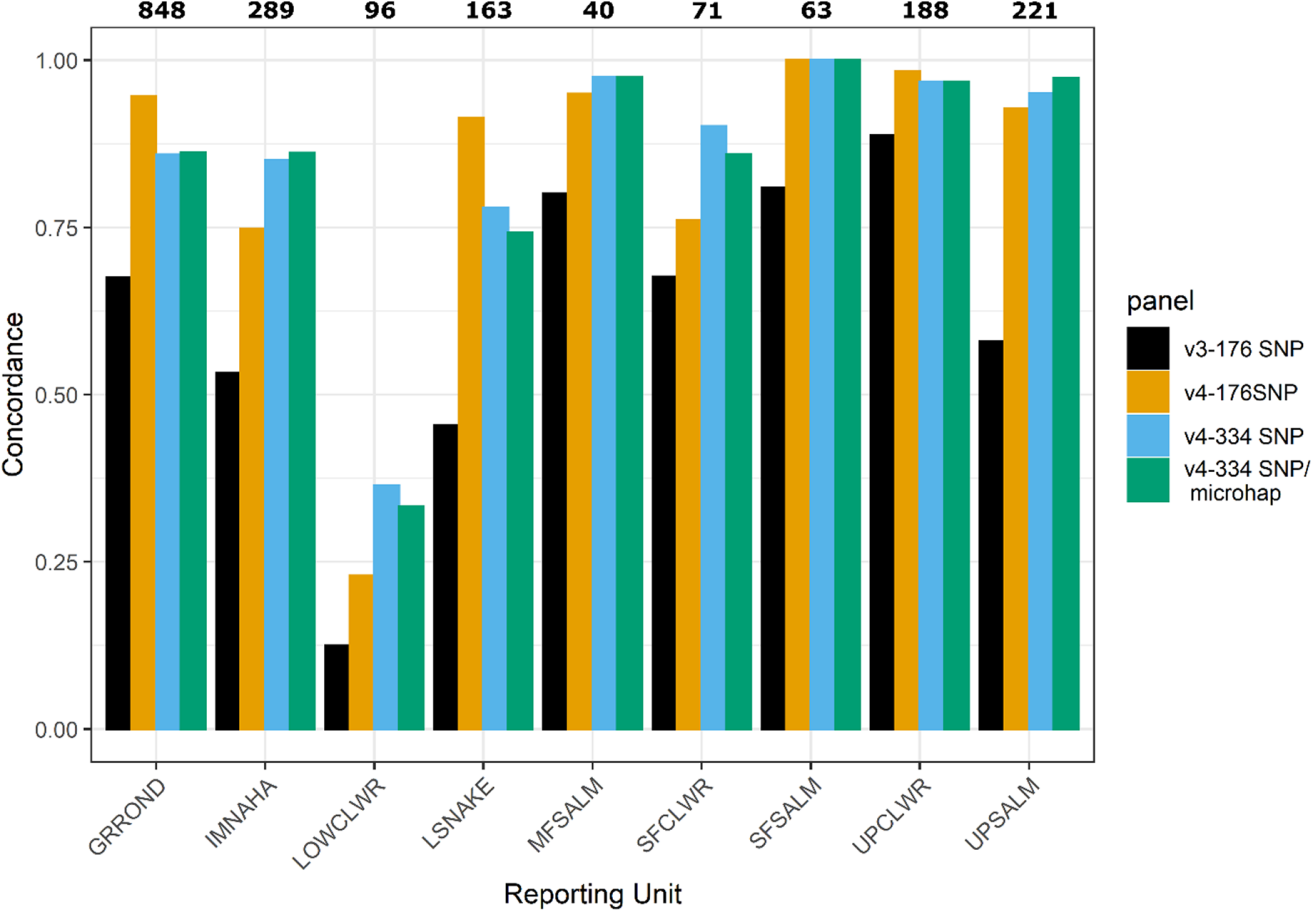
The proportion of PIT tag detections and GSI stock assignments that were concordant for adult steelhead (*Oncorhynchus mykiss*) returning to the Snake River basin for spawn years 2019 and 2020. Numbers in bold correspond to the number of PIT tag observations per reporting unit. For a description of reporting unit abbreviations, see Figure 2.

Overall, 62.6% of samples were assigned to the same reporting unit via PIT tag and GSI for the older baseline (v3‐176 SNP) which was significantly lower (χ^2^ = 329.84, df = 1, *p*‐value <0.001) relative to the new baseline (87.6% of samples concordant) with the same number of SNPs (v4‐176 SNP, Table 2, Figure 5). Changes in concordance between baseline versions evaluated with 176 SNPs were not equal across reporting units; the largest increases were observed for the lower Snake (+46.0%) and Upper Salmon (+34.8%) reporting units and the smallest gains were observed in the South Fork Clearwater (+8.5%) and Upper Clearwater River (+9.6%).

Overall, estimates of concordance were not significantly different for different marker panel combinations associated with baseline v4. Specifically, the concordance rate was 85.6% for v4 at 334 SNPs which was not significantly different than 87.6% observed for v4‐176 SNPs (χ^2^ = 3.31, df = 1, *p*‐value = 0.07). Lastly, no significant differences were observed in concordance rates for v4‐334 SNP/microhaplotype (85.5%) relative to v4‐334 SNPs (χ^2^ = 0, df = 1, *p*‐value = 1).

## 4 DISCUSSION

Genetic stock identification plays an important role in fisheries management, and to date, efforts to enhance the resolution of genetic stock assignments have involved increasing sample coverage (Beacham et al., 2010; Habicht et al., 2010), increasing marker numbers (Larson et al., 2014), and changing marker types (e.g., Hess et al., 2011; Narum et al., 2008). In the current study, we evaluated the impacts of population representation, marker number, and marker type on the performance and accuracy of genetic stock assignments for steelhead in the Snake River basin through three pairwise comparisons of four genetic baseline/marker combinations (1. “older, larger baseline”; 2. “newer, smaller baseline”; 3. “new baseline with more markers”; 4. “new baseline with microhaplotypes”). We observed significantly higher rates of self‐assignment for the “older, larger baseline” relative to the “newer, smaller” version and when more markers (“new baseline with more markers”) and marker types with higher variability (microhaplotypes) were included (“new baseline with microhaplotypes”). Overall, self‐assignment rates were highest for the “newer baseline with microhaplotypes.” Bias was smallest for the “older, larger baseline,” but this was not significantly different relative to the “newer, smaller baseline” surveyed at the same marker panel. We failed to observe significant differences in bias between the “new baseline with more markers” relative to the “new baseline with microhaplotypes,” despite both versions of these new baselines having equal numbers of loci. While assignment bias was lowest for the “older, larger baseline,” we note that bias was small and mixture estimates were unbiased regardless of baseline‐marker combination. Concordance rates between PIT tag detections and GSI assignment were higher for all three versions of the “newer baseline” regardless of marker number or type. While concordance was highest for the “newer, smaller baseline” at the smallest marker panel (176 SNPs), concordance rates varied little among the three marker‐number/type versions of the newer baseline. Combined, our study provides evidence that efficient representation of populations (i.e., fewer individuals from a smaller number of populations distributed more broadly across the landscape) can improve GSI assignments rates when marker number and/or informativeness is increased.

Self‐assignment rates to our reference baselines were significantly impacted by marker number and type, and may be explained by a better ability to differentiate between related reporting units through added informational content in additional and more variable markers (Table 3). Simulation work has shown that fewer markers were necessary to assign fish to their stock of origin when strongly differentiated (Kalinowski, 2004) and that error estimates of GSI decline exponentially with increasing mean pairwise *F*_ST_ values in genetic baselines (Araujo et al., 2014). Among populations of Coho salmon (*Oncorhynchus kisutch*) in western Canada, populations that were most genetically distinct showed the highest rates of self‐assignment (Beacham et al., 2020). In the case of steelhead in the Snake River basin, average per locus estimates of *F*_ST_ values were highest for “newer baseline with more markers” which was nearly identical to the “newer baseline with microhaplotypes,” followed by the “older, larger baseline,” and “newer, smaller baseline.” Average rates of self‐assignment were overall highest for the “newer baseline with microhaplotypes” followed by “newer baseline with more markers,” “older, larger baseline,” and “newer, smaller baseline.” At the individual stock level, we noted the largest increases in self‐assignment rates (“newer baseline with microhaplotypes” vs “older, larger baseline”) in reporting units (Grande Ronde, lower Snake, and upper Salmon rivers) which displayed lower levels of pairwise genetic differentiation and have been subject to hatchery supplementation (Hargrove, Delomas, et al., 2021; Nielsen et al., 2009). In contrast, increases in self‐assignment rates were lowest in reporting units managed for natural fish production (i.e., no hatchery supplementation; upper Clearwater and Middle Fork Salmon rivers) that displayed levels of genetic differentiation above basin‐wide averages (South Fork Clearwater River, Hargrove, Delomas, et al., 2021). The superior performance of our baseline containing microhaplotypes was promising and may similarly benefit other species which display complex patterns of genetic structure. To note, the markers added to our panel were designed for applications at the Columbia River basin scale, and future marker discovery efforts that target maximizing differentiation among Snake River basin stocks may yield greater increases in self‐assignment rates.

In addition to marker number and type, population representation (i.e., number of individuals per populations and the number of populations) significantly impacted self‐assignment rates, which was not necessarily expected. Research has shown the number of individuals sampled per population can affect the performance of genetic stock assignments (e.g., Beacham et al., 2010, 2020), with higher accuracy obtained with more individuals until a point of diminishing returns is reached. In the current study, we reduced the overall number of individuals genotyped in the newer baseline by nearly half (v3: *n* = 5967, v4: *n* = 3150), but expanded our sampling across the landscape (i.e., more management populations) which were represented by fewer populations (i.e., genetically similar groups of individuals) in the baseline. These differences in population representation between baselines resulted in higher self‐assignment rates for the “older, larger baseline” compared to the “newer, smaller baselines”; however, these differences varied by reporting unit and were inconsistent in direction. The number of samples per population in stock identification studies varies, but is commonly near 100 samples (e.g., Beacham et al., 2010; Narum et al., 2008; Smith et al., 2005; Smith & Seeb, 2008). While 80–100 samples can produce higher assignment accuracy (Beacham et al., 2010; Figure 2), losses in accuracy appear greatest for much smaller sample sizes (e.g., 20–40). In Snake River basin steelhead, the average number of individuals per population decreased between baselines (v3 = 133, v4 = 72), but this change alone seemed insufficient to explain the observed decrease in self‐assignment rates. The impacts of sample size on assignment rates have been shown to be strongly impacted by the distinctiveness of the population; highly differentiated populations require fewer samples (i.e., 40 individuals) to obtain similar accuracy as weakly differentiated ones (>250; Beacham et al., 2020). Morin et al. (2009) identified via simulations that approximately four times as many samples (20 vs 80 individuals) are required to obtain equivalent power when differentiation is weaker (*F*_ST_ = 0.0025 vs. *F*_ST_ = 0.01). In terms of added population coverage, our new baseline included two previously unsampled management populations (upper Grande Ronde River and Panther Creek), but the addition of these management populations did not appear to positively affect assignment rates. This may be a result of the fact that both the upper Grande Ronde River (GRUMA‐s) and Panther Creek (SRPAN‐s) exhibit lower than average genetic differentiation relative to other populations in the basin (Hargrove et al., 2023). While we were unable to disentangle the effects of sample size and sample source on self‐assignment rates, we note that both factors should be carefully considered when constructing new baselines or updating existing ones.

Attempts to quantify accuracy of genetic stock assignments via simulated mixtures revealed generally low error regardless of baseline, marker number, or marker type. Deviations between estimated and true mixture proportions were lowest for the “older, larger baseline” and among versions of the newer baseline, bias was lowest for the “newer, smaller baselines” and increased as marker number (“newer baseline with more markers”) and marker variability increased (“newer baseline with microhaplotypes”). This pattern was expected as previous work has demonstrated more samples are required to reduce sampling variation in allele frequency estimates of more variable markers (e.g., microsatellites vs. bi‐allelic SNPs; Beacham et al., 2010). That bias did not differ significantly between the “newer baseline with more markers” relative to the “newer baseline with microhaplotypes” may be the modest number of alleles per locus (up to 6, with 66% of loci containing three alleles) at the relatively small number of microhaplotype loci (91). For markers such as microsatellites which can average 30 alleles per locus (Beacham et al., 2010) more samples are needed to accurately estimate allele frequencies within populations. Importantly, the low overall bias observed for the “older, larger baseline” as well as the version of the newer baseline containing both SNPs and microhaplotypes implies that estimates of stock composition and abundance generated for returning adult steelhead in the Snake River basin are accurate.

A common practice when establishing GSI baselines is to parse samples into training and holdout sets to avoid “high‐grading bias”, which occurs when the resolution of loci is biased because the same set of samples were used to establish and evaluate baseline performance (e.g., Clemento et al., 2014; May et al., 2020). In our case, we used a separate collection of returning adult steelhead that were genetically sampled and PIT‐tagged prior to entering the Snake River basin. These individuals were subsequently detected at in‐stream PIT tag detection arrays on the landscape and their last known physical location was compared with their GSI assignment. As noted previously, we assumed that highest concordance between PIT‐last‐known‐location with GSI assignment could be used as a proxy for highest accuracy of a GSI baseline because natural‐origin steelhead in the Snake River basin display high natal‐stream fidelity (Keefer & Caudill, 2012). Results from these analyses indicated that population representation but not marker panel/marker type had the greatest impact on concordance, and we argue this impact was driven by broader population coverage associated with the newer baseline. Specifically, baseline v4 had fewer genetic populations (44) but more management populations (24) relative to baseline v3 (45 genetic populations, 22 management populations). That we observed increased concordance despite having fewer total individuals and populations in the new baseline implies some collections in the older baseline may have been redundant or were poorly differentiated at the assayed markers. Changes in concordance rates among reporting units were not equal and select groups had high assignment rates regardless of marker panel (e.g., Upper Clearwater River, South Fork Salmon River, Upper Salmon River). In general, concordance was highest for reporting groups that displayed above average levels of genetic differentiation, were minimally influenced by hatchery supplementation, and were located at terminal (uppermost) portions of river drainages (Nielsen et al., 2009; Campbell et al., 2012; Powell & Campbell, 2020). One reason why concordance may be more limited in downstream reporting groups is the inherent difficulty associated with installing in‐stream PIT tag arrays in lower elevation, higher flow portions of drainage basins. In the Snake River basin, we were unable to estimate concordance for the lower Salmon River reporting unit as this reporting unit is characterized by a large main‐stem river (i.e., flows too high for PIT tag array operation) and large numbers of small tributary creeks. It is also important to point out that physical tagging (last known location) and genetic data (genetic reporting unit of origin) quantify fundamentally different aspects of steelhead biology, and it is entirely possible that fish can stray on the landscape and fail to reproduce in the area where they were last observed. Despite these limitations, relating PIT tag derived location data to genetic stock assignments represents an additional opportunity to validate baseline performance and the pairing of genetic and PIT tag data have been instrumental salmonid research, including efforts to identify the genetic basis of life history characteristics (e.g., Micheletti et al., 2018; Willis et al., 2020, 2021).

## 5 CONCLUSIONS

The current study sought to use the description of a new genetic baseline as a case study to understand how population representation, marker number, and marker type affected the performance of genetic stock identification methods. We showed that a smaller number of individuals that are efficiently distributed to represent all existing populations can be genotyped at a combination of SNPs and microhaplotypes and produce higher rates of self‐assignment relative to a larger baseline typed at fewer markers. Associated reductions in sample processing results in a substantial reduction in costs which is particularly important for ongoing monitoring programs that benefit from periodic updates to genetic baselines. The current study was not without its limitations, and it is important to note that the additional SNPs and microhaplotypes added to our marker panel were not specifically designed to maximize differentiation among Snake River basin stocks of steelhead, but instead were adopted to maintain marker consistency with monitoring efforts of stocks at the Columbia River basin scale. Importantly, the discovery of microhaplotypes within the given marker panel was done opportunistically via modification of bioinformatic processing and yielded additional resolution for genetic stock identification purposes at no additional cost. The use of microhaplotypes has expanded considerably in recent years, and we add to the growing list of studies which have shown the utility of this marker type in a wide range of applications (e.g., identify sources of fisheries bycatch, Baetscher et al., 2022; quantify relative composition in pooled DNA samples Shi et al., 2023; identify grandparent–grandchild trios, Delomas et al., 2021; and describe differentiate populations in marine mammals with weak genetic structure, Morin et al., 2021). Moving forward, we expect the use of microhaplotypes in GSI related studies to expand both given their ability to help resolve weakly differentiated stocks. In the Snake River basin efforts are underway to create a new, larger marker panel consisting entirely of microhaplotypes specific to steelhead to potentially resolve ambiguous or poorly assigning reporting units.

## CONFLICT OF INTEREST STATEMENT

The authors have no conflict of interests related to this publication.

## Supporting information


File S1.

File S2.

File S3.

File S4.

File S5.

File S6.

## Data Availability

Genetic data used as part of this research has been provided as supplemental files.
