# Supplementary material for: Efficient population representation with more genetic markers increases performance of a steelhead (Oncorhynchus mykiss) genetic stock identification baseline
Source: Evol Appl. 2023 Dec 26;17(2):e13610. doi: 10.1111/eva.13610 (PMC10853585; doi:10.1111/eva.13610)
Supplement: Supplementary file 2 — File S2. [file EVA-17-e13610-s005.docx]

Supplemental File . A list of single nucleotide polymorphisms (SNPs) used for the construction of genetic baseline version 4 (v4) for steelhead (*Oncorhynchus mykiss*) in the Snake River Basin, USA. For each marker we provide a description of the marker type (microhaplotype or bi-allelic SNP), number of alleles (N_A_), expected heterozygosity (H_E_), observed heterozygosity (H_O_), and fixation index (F_ST_) based on collections from the Snake River basin GSI baseline v4. A copy of the genetic baseline can be downloaded at [fishgen.net](https://www.fishgen.net/) by searching fishgen ID = ‘Snake River steelhead GSI baseline version 4’.

| **Marker** | **type** | **N_A_** | **H_E_** | **H_O_** | **F_ST_** |
| --- | --- | --- | --- | --- | --- |
| OMS00039 | microhaplotype | 2.43 | 0.506 | 0.503 | 0.001 |
| OMS00052 | microhaplotype | 3 | 0.633 | 0.611 | 0.023 |
| OMS00077 | microhaplotype | 2.43 | 0.488 | 0.464 | 0.031 |
| OMS00101 | microhaplotype | 2.27 | 0.495 | 0.476 | 0.033 |
| OMS00116 | microhaplotype | 2.09 | 0.1 | 0.1 | 0.034 |
| OMS00118 | microhaplotype | 2.75 | 0.505 | 0.487 | 0.051 |
| OMS00120 | microhaplotype | 2.82 | 0.32 | 0.304 | 0.048 |
| OMS00128 | microhaplotype | 2.16 | 0.079 | 0.077 | 0.024 |
| OMS00129 | microhaplotype | 3.89 | 0.61 | 0.601 | 0.025 |
| OMS00143 | microhaplotype | 3.23 | 0.221 | 0.215 | 0.032 |
| OMS00149 | microhaplotype | 2.84 | 0.545 | 0.522 | 0.047 |
| OMS00151 | microhaplotype | 2.27 | 0.296 | 0.287 | 0.03 |
| OMS00175 | microhaplotype | 2.41 | 0.494 | 0.48 | 0.024 |
| Omy_101832-195 | microhaplotype | 2.14 | 0.489 | 0.472 | 0.026 |
| Omy_102867-443 | microhaplotype | 1.8 | 0.069 | 0.067 | 0.027 |
| Omy_104519-624 | microhaplotype | 2.11 | 0.448 | 0.433 | 0.047 |
| Omy_107336-170 | microhaplotype | 2.66 | 0.279 | 0.27 | 0.046 |
| Omy_108007-193 | microhaplotype | 2.11 | 0.475 | 0.454 | 0.033 |
| Omy_109243-222 | microhaplotype | 2.98 | 0.371 | 0.361 | 0.019 |
| Omy_110201-359 | microhaplotype | 3.09 | 0.479 | 0.447 | 0.066 |
| Omy_111383-51 | microhaplotype | 2.5 | 0.498 | 0.494 | 0.023 |
| Omy_112301-202 | microhaplotype | 1.75 | 0.058 | 0.058 | 0.028 |
| Omy_112820-82 | microhaplotype | 2.11 | 0.067 | 0.065 | 0.022 |
| Omy_117540-259 | microhaplotype | 2.3 | 0.231 | 0.213 | 0.055 |
| Omy_120255-332 | microhaplotype | 1.8 | 0.06 | 0.056 | 0.031 |
| Omy_128923-433 | microhaplotype | 2.39 | 0.507 | 0.48 | 0.029 |
| Omy_130524-160 | microhaplotype | 2.59 | 0.479 | 0.463 | 0.014 |
| Omy_131460-646 | microhaplotype | 1.89 | 0.045 | 0.044 | 0.028 |
| Omy_187760-385 | microhaplotype | 1.5 | 0.025 | 0.024 | 0.021 |
| Omy_96222-125 | microhaplotype | 2.89 | 0.319 | 0.318 | 0.027 |
| Omy_97077-73 | microhaplotype | 2.43 | 0.226 | 0.212 | 0.058 |
| Omy_97954-618 | microhaplotype | 2.68 | 0.365 | 0.348 | 0.035 |
| Omy_99300-202 | microhaplotype | 2.75 | 0.377 | 0.375 | 0.027 |
| Omy_anp-17 | microhaplotype | 2.98 | 0.501 | 0.467 | 0.074 |
| Omy_aromat-280 | microhaplotype | 2.86 | 0.354 | 0.355 | 0.017 |
| Omy_aspAT-123 | microhaplotype | 2.46 | 0.411 | 0.405 | 0.025 |
| Omy_BAC-F5.284 | microhaplotype | 2.8 | 0.43 | 0.422 | 0.027 |
| Omy_ca050-64 | microhaplotype | 2.84 | 0.475 | 0.46 | 0.022 |
| Omy_carban1-264 | microhaplotype | 2.82 | 0.206 | 0.204 | 0.032 |
| Omy_cox2-335 | microhaplotype | 2.93 | 0.362 | 0.348 | 0.022 |
| Omy_cyp17-153 | microhaplotype | 2.39 | 0.203 | 0.17 | 0.062 |
| Omy_e1-147 | microhaplotype | 2.07 | 0.086 | 0.085 | 0.016 |
| Omy_g12-82 | microhaplotype | 2.11 | 0.499 | 0.494 | 0.027 |
| Omy_GHSR-121 | microhaplotype | 2.8 | 0.292 | 0.271 | 0.048 |
| Omy_hsc715-80 | microhaplotype | 3.16 | 0.551 | 0.509 | 0.041 |
| Omy_IL1b-163 | microhaplotype | 2.57 | 0.238 | 0.222 | 0.078 |
| Omy_lpl-220 | microhaplotype | 3.55 | 0.447 | 0.437 | 0.034 |
| Omy_mcsf-268 | microhaplotype | 2.16 | 0.107 | 0.106 | 0.047 |
| Omy_metA-161 | microhaplotype | 2.48 | 0.402 | 0.386 | 0.035 |
| Omy_metB-138 | microhaplotype | 3.02 | 0.327 | 0.322 | 0.021 |
| Omy_MYC_2 | microhaplotype | 2.25 | 0.429 | 0.423 | 0.012 |
| Omy_nach-200 | microhaplotype | 1.96 | 0.065 | 0.058 | 0.043 |
| Omy_nkef-241 | microhaplotype | 2.14 | 0.487 | 0.473 | 0.021 |
| Omy_nxt2-273 | microhaplotype | 2.93 | 0.204 | 0.198 | 0.035 |
| Omy_oxct-85 | microhaplotype | 2.68 | 0.229 | 0.225 | 0.04 |
| Omy_pad-196 | microhaplotype | 2.89 | 0.14 | 0.134 | 0.022 |
| Omy_ppie-232 | microhaplotype | 2.11 | 0.215 | 0.205 | 0.014 |
| Omy_RAD16104-20 | microhaplotype | 2.96 | 0.558 | 0.553 | 0.012 |
| Omy_RAD18903-48 | microhaplotype | 1.84 | 0.075 | 0.073 | 0.028 |
| Omy_RAD23894-58 | microhaplotype | 3.46 | 0.585 | 0.541 | 0.022 |
| Omy_RAD2567-8 | microhaplotype | 3.36 | 0.582 | 0.556 | 0.053 |
| Omy_RAD26080-69 | microhaplotype | 2.5 | 0.502 | 0.474 | 0.079 |
| Omy_RAD26691-36 | microhaplotype | 2.91 | 0.455 | 0.452 | 0.021 |
| Omy_RAD29700-18 | microhaplotype | 2.52 | 0.365 | 0.343 | 0.028 |
| Omy_RAD2976-26 | microhaplotype | 2.48 | 0.184 | 0.174 | 0.052 |
| Omy_RAD3926-22 | microhaplotype | 2 | 0.204 | 0.193 | 0.08 |
| Omy_RAD43612-42 | microhaplotype | 3 | 0.656 | 0.637 | 0.03 |
| Omy_RAD47444-53 | microhaplotype | 2.93 | 0.363 | 0.358 | 0.033 |
| Omy_RAD49111-35 | microhaplotype | 2.96 | 0.536 | 0.519 | 0.041 |
| Omy_RAD52458-17 | microhaplotype | 2.11 | 0.227 | 0.158 | 0.309 |
| Omy_RAD52812-28 | microhaplotype | 2.39 | 0.447 | 0.407 | 0.034 |
| Omy_RAD5374-56 | microhaplotype | 2.96 | 0.238 | 0.201 | 0.04 |
| Omy_RAD58213-70 | microhaplotype | 3.32 | 0.351 | 0.344 | 0.024 |
| Omy_RAD58835-15 | microhaplotype | 2.16 | 0.29 | 0.272 | 0.037 |
| Omy_RAD7016-31 | microhaplotype | 3 | 0.667 | 0.658 | 0.025 |
| Omy_RAD7210-8 | microhaplotype | 3.07 | 0.543 | 0.526 | 0.032 |
| Omy_RAD72528-44 | microhaplotype | 1.77 | 0.139 | 0.124 | 0.051 |
| Omy_RAD73204-63 | microhaplotype | 2.86 | 0.178 | 0.172 | 0.024 |
| Omy_RAD7384-50 | microhaplotype | 1.91 | 0.073 | 0.07 | 0.05 |
| Omy_RAD73963-73 | microhaplotype | 3.91 | 0.572 | 0.554 | 0.043 |
| Omy_RAD76570-62 | microhaplotype | 2.23 | 0.113 | 0.112 | 0.031 |
| Omy_RAD79314-58 | microhaplotype | 1.34 | 0.013 | 0.013 | 0.018 |
| Omy_RAD86706-72 | microhaplotype | 2.27 | 0.457 | 0.412 | 0.155 |
| Omy_RAD9004-13 | microhaplotype | 2.91 | 0.556 | 0.544 | 0.028 |
| Omy_RAD92485-64 | microhaplotype | 4.41 | 0.557 | 0.509 | 0.04 |
| Omy_sast-264 | microhaplotype | 2.16 | 0.307 | 0.303 | 0.018 |
| Omy_SECC22b-88 | microhaplotype | 2.39 | 0.203 | 0.2 | 0.047 |
| Omy_sys1-188 | microhaplotype | 2.48 | 0.231 | 0.222 | 0.047 |
| Omy_tlr5-205 | microhaplotype | 2.36 | 0.152 | 0.147 | 0.028 |
| Omy_txnip-343 | microhaplotype | 2.57 | 0.394 | 0.379 | 0.028 |
| Omy_u09-56.119 | microhaplotype | 3.43 | 0.255 | 0.245 | 0.029 |
| Omy_U11_2b-154 | microhaplotype | 3.39 | 0.459 | 0.455 | 0.025 |
| M09AAC.055 | biallelic SNP | 2 | 0.129 | 0.127 | 0.036 |
| M09AAD.076 | biallelic SNP | 2 | 0.499 | 0.476 | 0.02 |
| M09AAE.082 | biallelic SNP | 2 | 0.317 | 0.301 | 0.022 |
| M09AAJ.163 | biallelic SNP | 2 | 0.437 | 0.41 | 0.035 |
| OMGH1PROM1-SNP1 | biallelic SNP | 1.98 | 0.175 | 0.165 | 0.063 |
| OMS00002 | biallelic SNP | 2 | 0.466 | 0.442 | 0.019 |
| OMS00003 | biallelic SNP | 2 | 0.265 | 0.257 | 0.022 |
| OMS00006 | biallelic SNP | 2 | 0.499 | 0.495 | 0.031 |
| OMS00008 | biallelic SNP | 2 | 0.288 | 0.269 | 0.04 |
| OMS00013 | biallelic SNP | 1.98 | 0.156 | 0.151 | 0.024 |
| OMS00014 | biallelic SNP | 1.48 | 0.041 | 0.041 | 0.029 |
| OMS00015 | biallelic SNP | 1.98 | 0.105 | 0.1 | 0.031 |
| OMS00017 | biallelic SNP | 2 | 0.389 | 0.351 | 0.066 |
| OMS00018 | biallelic SNP | 2 | 0.181 | 0.18 | 0.031 |
| OMS00024 | biallelic SNP | 2 | 0.465 | 0.448 | 0.039 |
| OMS00030 | biallelic SNP | 1.98 | 0.165 | 0.161 | 0.023 |
| OMS00041 | biallelic SNP | 1.5 | 0.039 | 0.036 | 0.04 |
| OMS00048 | biallelic SNP | 2 | 0.197 | 0.187 | 0.028 |
| OMS00053 | biallelic SNP | 2 | 0.493 | 0.452 | 0.024 |
| OMS00056 | biallelic SNP | 2 | 0.343 | 0.333 | 0.026 |
| OMS00057 | biallelic SNP | 2 | 0.462 | 0.431 | 0.045 |
| OMS00058 | biallelic SNP | 2 | 0.488 | 0.445 | 0.057 |
| OMS00061 | biallelic SNP | 1.86 | 0.126 | 0.119 | 0.035 |
| OMS00062 | biallelic SNP | 2 | 0.38 | 0.38 | 0.02 |
| OMS00064 | biallelic SNP | 2 | 0.471 | 0.433 | 0.049 |
| OMS00068 | biallelic SNP | 2 | 0.437 | 0.432 | 0.021 |
| OMS00070 | biallelic SNP | 2 | 0.5 | 0.488 | 0.035 |
| OMS00071 | biallelic SNP | 2 | 0.487 | 0.467 | 0.022 |
| OMS00072 | biallelic SNP | 2 | 0.494 | 0.485 | 0.021 |
| OMS00074 | biallelic SNP | 2 | 0.493 | 0.444 | 0.062 |
| OMS00078 | biallelic SNP | 2 | 0.394 | 0.382 | 0.022 |
| OMS00079 | biallelic SNP | 2 | 0.5 | 0.489 | 0.028 |
| OMS00087 | biallelic SNP | 2 | 0.288 | 0.234 | 0.025 |
| OMS00089 | biallelic SNP | 2 | 0.4 | 0.376 | 0.038 |
| OMS00090 | biallelic SNP | 2 | 0.5 | 0.48 | 0.047 |
| OMS00092 | biallelic SNP | 2 | 0.267 | 0.244 | 0.072 |
| OMS00095 | biallelic SNP | 1.89 | 0.12 | 0.111 | 0.051 |
| OMS00096 | biallelic SNP | 2 | 0.327 | 0.324 | 0.017 |
| OMS00103 | biallelic SNP | 1.5 | 0.034 | 0.032 | 0.012 |
| OMS00105 | biallelic SNP | 2 | 0.462 | 0.452 | 0.027 |
| OMS00106 | biallelic SNP | 2 | 0.355 | 0.336 | 0.038 |
| OMS00111 | biallelic SNP | 2 | 0.32 | 0.296 | 0.047 |
| OMS00112 | biallelic SNP | 1.98 | 0.324 | 0.307 | 0.039 |
| OMS00114 | biallelic SNP | 2 | 0.161 | 0.158 | 0.023 |
| OMS00119 | biallelic SNP | 2 | 0.239 | 0.231 | 0.024 |
| OMS00121 | biallelic SNP | 2 | 0.497 | 0.49 | 0.016 |
| OMS00127 | biallelic SNP | 1.3 | 0.009 | 0.009 | 0.012 |
| OMS00132 | biallelic SNP | 2 | 0.486 | 0.468 | 0.019 |
| OMS00133 | biallelic SNP | 1.55 | 0.041 | 0.038 | 0.029 |
| OMS00134 | biallelic SNP | 1.5 | 0.038 | 0.036 | 0.036 |
| OMS00138 | biallelic SNP | 2 | 0.194 | 0.185 | 0.03 |
| OMS00153 | biallelic SNP | 1.91 | 0.126 | 0.123 | 0.021 |
| OMS00154 | biallelic SNP | 2 | 0.334 | 0.318 | 0.027 |
| OMS00156 | biallelic SNP | 2 | 0.448 | 0.434 | 0.025 |
| OMS00164 | biallelic SNP | 1.48 | 0.029 | 0.029 | 0.011 |
| OMS00169 | biallelic SNP | 1.36 | 0.026 | 0.026 | 0.023 |
| OMS00173 | biallelic SNP | 2 | 0.226 | 0.222 | 0.017 |
| OMS00174 | biallelic SNP | 1.98 | 0.089 | 0.089 | 0.008 |
| OMS00176 | biallelic SNP | 1.84 | 0.129 | 0.125 | 0.03 |
| OMS00179 | biallelic SNP | 2 | 0.409 | 0.348 | 0.039 |
| OMS00180 | biallelic SNP | 2 | 0.458 | 0.41 | 0.045 |
| Omy_1004 | biallelic SNP | 2 | 0.298 | 0.285 | 0.02 |
| Omy_101554-306 | biallelic SNP | 1.86 | 0.115 | 0.117 | 0.035 |
| Omy_101993-189 | biallelic SNP | 2 | 0.374 | 0.363 | 0.039 |
| Omy_102505-102 | biallelic SNP | 2 | 0.46 | 0.445 | 0.02 |
| Omy_103705-558 | biallelic SNP | 1.98 | 0.195 | 0.187 | 0.019 |
| Omy_105075-162 | biallelic SNP | 1.98 | 0.164 | 0.158 | 0.019 |
| Omy_105105-448 | biallelic SNP | 2 | 0.494 | 0.457 | 0.044 |
| Omy_105385-406 | biallelic SNP | 2 | 0.473 | 0.45 | 0.024 |
| Omy_105714-265 | biallelic SNP | 2 | 0.418 | 0.402 | 0.02 |
| Omy_107031-704 | biallelic SNP | 2 | 0.282 | 0.274 | 0.024 |
| Omy_107285-69 | biallelic SNP | 2 | 0.277 | 0.271 | 0.012 |
| Omy_107806-34 | biallelic SNP | 2 | 0.442 | 0.422 | 0.047 |
| Omy_109525-403 | biallelic SNP | 2 | 0.415 | 0.41 | 0.015 |
| Omy_109894-185 | biallelic SNP | 2 | 0.453 | 0.377 | 0.035 |
| Omy_110064-419 | biallelic SNP | 2 | 0.474 | 0.452 | 0.042 |
| Omy_110362-585 | biallelic SNP | 1.48 | 0.023 | 0.023 | 0.012 |
| Omy_110689-148 | biallelic SNP | 2 | 0.255 | 0.247 | 0.025 |
| Omy_111084-526 | biallelic SNP | 2 | 0.332 | 0.331 | 0.018 |
| Omy_111666-301 | biallelic SNP | 1.57 | 0.051 | 0.048 | 0.042 |
| Omy_113490-159 | biallelic SNP | 2 | 0.484 | 0.439 | 0.063 |
| Omy_114315-438 | biallelic SNP | 2 | 0.489 | 0.423 | 0.066 |
| Omy_114587-480 | biallelic SNP | 2 | 0.453 | 0.426 | 0.037 |
| Omy_114976-223 | biallelic SNP | 2 | 0.475 | 0.352 | 0.037 |
| Omy_116733-349 | biallelic SNP | 2 | 0.419 | 0.411 | 0.02 |
| Omy_116938-264 | biallelic SNP | 1.82 | 0.119 | 0.115 | 0.043 |
| Omy_117286-374 | biallelic SNP | 1.93 | 0.154 | 0.153 | 0.016 |
| Omy_117370-400 | biallelic SNP | 2 | 0.497 | 0.462 | 0.022 |
| Omy_117815-81 | biallelic SNP | 1.98 | 0.413 | 0.378 | 0.086 |
| Omy_118175-396 | biallelic SNP | 1.27 | 0.012 | 0.011 | 0.013 |
| Omy_118654-91 | biallelic SNP | 2 | 0.364 | 0.347 | 0.035 |
| Omy_128693-455 | biallelic SNP | 1.09 | 0.01 | 0.008 | 0.013 |
| Omy_128996-481 | biallelic SNP | 2 | 0.15 | 0.119 | 0.019 |
| Omy_129870-756 | biallelic SNP | 2 | 0.283 | 0.255 | 0.02 |
| Omy_97660-230 | biallelic SNP | 2 | 0.459 | 0.448 | 0.03 |
| Omy_97865-196 | biallelic SNP | 1.8 | 0.089 | 0.085 | 0.02 |
| Omy_98683-165 | biallelic SNP | 1.8 | 0.08 | 0.074 | 0.069 |
| Omy_ada10-71 | biallelic SNP | 2 | 0.311 | 0.304 | 0.008 |
| Omy_arp-630 | biallelic SNP | 2 | 0.496 | 0.478 | 0.02 |
| Omy_b1-266 | biallelic SNP | 2 | 0.398 | 0.383 | 0.01 |
| Omy_b9-164 | biallelic SNP | 1.98 | 0.204 | 0.159 | 0.102 |
| Omy_BAC-B4-324 | biallelic SNP | 2 | 0.497 | 0.478 | 0.032 |
| Omy_BAMBI2.312 | biallelic SNP | 1.93 | 0.183 | 0.178 | 0.029 |
| Omy_BAMBI4.238 | biallelic SNP | 1.07 | 0.002 | 0.002 | 0.011 |
| Omy_bcAKala-380rd | biallelic SNP | 2 | 0.435 | 0.43 | 0.03 |
| Omy_cd28-130 | biallelic SNP | 1.66 | 0.034 | 0.033 | 0.006 |
| Omy_cd59-206 | biallelic SNP | 2 | 0.415 | 0.408 | 0.015 |
| Omy_cd59b-112 | biallelic SNP | 1.91 | 0.197 | 0.144 | 0.044 |
| Omy_cin-172 | biallelic SNP | 2 | 0.335 | 0.318 | 0.03 |
| Omy_cox1-221 | biallelic SNP | 2 | 0.481 | 0.458 | 0.037 |
| Omy_crb-106 | biallelic SNP | 2 | 0.483 | 0.398 | 0.067 |
| Omy_ftzf1-217 | biallelic SNP | 1.27 | 0.017 | 0.017 | 0.011 |
| Omy_g1-103 | biallelic SNP | 1.75 | 0.126 | 0.12 | 0.036 |
| Omy_G3PD_2.246 | biallelic SNP | 1.3 | 0.016 | 0.017 | 0.031 |
| Omy_G3PD_2-371 | biallelic SNP | 2 | 0.285 | 0.275 | 0.029 |
| Omy_gadd45-332 | biallelic SNP | 2 | 0.199 | 0.189 | 0.067 |
| Omy_gdh-271 | biallelic SNP | 2 | 0.185 | 0.17 | 0.04 |
| Omy_GH1P1_2 | biallelic SNP | 1.48 | 0.036 | 0.03 | 0.028 |
| Omy_gh-475 | biallelic SNP | 2 | 0.234 | 0.226 | 0.021 |
| Omy_gluR-79 | biallelic SNP | 2 | 0.498 | 0.49 | 0.019 |
| Omy_hsf1b-241 | biallelic SNP | 1.96 | 0.147 | 0.147 | 0.017 |
| Omy_hsf2-146 | biallelic SNP | 2 | 0.441 | 0.409 | 0.069 |
| Omy_hsp47-86 | biallelic SNP | 2 | 0.339 | 0.339 | 0.012 |
| Omy_hsp70aPro-329 | biallelic SNP | 1.91 | 0.095 | 0.085 | 0.065 |
| Omy_hsp90BA-193 | biallelic SNP | 1.86 | 0.131 | 0.118 | 0.037 |
| Omy_hus1-52 | biallelic SNP | 1.71 | 0.115 | 0.108 | 0.028 |
| Omy_IL17-185 | biallelic SNP | 2 | 0.497 | 0.501 | 0.026 |
| Omy_Il-1b_.028 | biallelic SNP | 2 | 0.297 | 0.29 | 0.035 |
| Omy_IL6-320 | biallelic SNP | 2 | 0.352 | 0.345 | 0.021 |
| Omy_impa1-55 | biallelic SNP | 2 | 0.136 | 0.128 | 0.024 |
| Omy_inos-97 | biallelic SNP | 1.98 | 0.102 | 0.096 | 0.027 |
| Omy_LDHB-1_i2 | biallelic SNP | 1.96 | 0.145 | 0.134 | 0.015 |
| Omy_LDHB-2_e5 | biallelic SNP | 2 | 0.277 | 0.275 | 0.007 |
| Omy_LDHB-2_i6 | biallelic SNP | 1.46 | 0.027 | 0.025 | 0.02 |
| Omy_mapK3-103 | biallelic SNP | 1.59 | 0.064 | 0.061 | 0.043 |
| Omy_myoD-178 | biallelic SNP | 1.91 | 0.21 | 0.197 | 0.03 |
| Omy_NaKATPa3-50 | biallelic SNP | 2 | 0.422 | 0.413 | 0.031 |
| Omy_ndk-152 | biallelic SNP | 1.71 | 0.061 | 0.061 | 0.021 |
| Omy_nips-299 | biallelic SNP | 1.96 | 0.119 | 0.115 | 0.013 |
| Omy_ntl-27 | biallelic SNP | 2 | 0.459 | 0.441 | 0.04 |
| Omy_Ogo4-212 | biallelic SNP | 2 | 0.485 | 0.461 | 0.028 |
| Omy_OmyP9-180 | biallelic SNP | 2 | 0.195 | 0.18 | 0.018 |
| Omy_Ots249-227 | biallelic SNP | 2 | 0.428 | 0.424 | 0.017 |
| Omy_p53-262 | biallelic SNP | 2 | 0.339 | 0.329 | 0.032 |
| Omy_RAD103359-45 | biallelic SNP | 1.05 | 0.001 | 0.001 | 0 |
| Omy_RAD10733-10 | biallelic SNP | 1.57 | 0.037 | 0.036 | 0.016 |
| Omy_RAD116-59 | biallelic SNP | 1.55 | 0.037 | 0.035 | 0.039 |
| Omy_RAD1186-59 | biallelic SNP | 2 | 0.422 | 0.413 | 0.034 |
| Omy_RAD12439-64 | biallelic SNP | 2 | 0.464 | 0.436 | 0.026 |
| Omy_RAD12566-14 | biallelic SNP | 1.21 | 0.003 | 0.003 | -0 |
| Omy_RAD13034-67 | biallelic SNP | 2 | 0.268 | 0.256 | 0.021 |
| Omy_RAD13073-16 | biallelic SNP | 1.82 | 0.086 | 0.082 | 0.025 |
| Omy_RAD13499-13 | biallelic SNP | 1.93 | 0.135 | 0.132 | 0.039 |
| Omy_RAD14033-46 | biallelic SNP | 1.84 | 0.069 | 0.068 | 0.013 |
| Omy_RAD15709-53 | biallelic SNP | 1.89 | 0.4 | 0.252 | 0.337 |
| Omy_RAD17632-23 | biallelic SNP | 2 | 0.434 | 0.426 | 0.022 |
| Omy_RAD17849-16 | biallelic SNP | 2 | 0.498 | 0.496 | 0.02 |
| Omy_RAD1919-22 | biallelic SNP | 1.21 | 0.006 | 0.006 | 0.009 |
| Omy_RAD19340-24 | biallelic SNP | 1.71 | 0.075 | 0.074 | 0.026 |
| Omy_RAD19578-59 | biallelic SNP | 2 | 0.186 | 0.136 | 0.078 |
| Omy_RAD20917-11 | biallelic SNP | 2 | 0.215 | 0.178 | 0.044 |
| Omy_RAD22123-69 | biallelic SNP | 2 | 0.478 | 0.461 | 0.045 |
| Omy_RAD23577-43 | biallelic SNP | 1.11 | 0.003 | 0.003 | 0.011 |
| Omy_RAD24287-74 | biallelic SNP | 2 | 0.372 | 0.343 | 0.064 |
| Omy_RAD25042-68 | biallelic SNP | 1.27 | 0.012 | 0.011 | 0.009 |
| Omy_RAD27740-55 | biallelic SNP | 1.77 | 0.08 | 0.069 | 0.023 |
| Omy_RAD28236-38 | biallelic SNP | 2 | 0.418 | 0.354 | 0.133 |
| Omy_RAD30392-17 | biallelic SNP | 2 | 0.408 | 0.337 | 0.049 |
| Omy_RAD30619-61 | biallelic SNP | 1.84 | 0.095 | 0.091 | 0.021 |
| Omy_RAD31408-67 | biallelic SNP | 2 | 0.476 | 0.415 | 0.104 |
| Omy_RAD3209-10 | biallelic SNP | 2 | 0.487 | 0.447 | 0.06 |
| Omy_RAD32139-58 | biallelic SNP | 2 | 0.316 | 0.293 | 0.08 |
| Omy_RAD33122-47 | biallelic SNP | 2 | 0.42 | 0.269 | 0.047 |
| Omy_RAD33798-24 | biallelic SNP | 1.91 | 0.124 | 0.119 | 0.035 |
| Omy_RAD35005-13 | biallelic SNP | 1.89 | 0.06 | 0.055 | 0.022 |
| Omy_RAD35149-9 | biallelic SNP | 2 | 0.391 | 0.371 | 0.041 |
| Omy_RAD35417-9 | biallelic SNP | 1.96 | 0.185 | 0.172 | 0.057 |
| Omy_RAD3651-48 | biallelic SNP | 2 | 0.484 | 0.398 | 0.024 |
| Omy_RAD366-7 | biallelic SNP | 2 | 0.43 | 0.396 | 0.044 |
| Omy_RAD36848-7 | biallelic SNP | 2 | 0.5 | 0.419 | 0.059 |
| Omy_RAD36952-53 | biallelic SNP | 1.59 | 0.103 | 0.098 | 0.049 |
| Omy_RAD37816-68 | biallelic SNP | 2 | 0.339 | 0.328 | 0.022 |
| Omy_RAD38406-19 | biallelic SNP | 1.98 | 0.155 | 0.1 | 0.035 |
| Omy_RAD39156-33 | biallelic SNP | 2 | 0.269 | 0.259 | 0.026 |
| Omy_RAD40132-55 | biallelic SNP | 2 | 0.452 | 0.435 | 0.027 |
| Omy_RAD40520-48 | biallelic SNP | 2 | 0.238 | 0.187 | 0.025 |
| Omy_RAD40641-58 | biallelic SNP | 1.89 | 0.109 | 0.104 | 0.047 |
| Omy_RAD41594-34 | biallelic SNP | 1.43 | 0.027 | 0.023 | 0.02 |
| Omy_RAD42465-32 | biallelic SNP | 1.8 | 0.047 | 0.045 | 0.017 |
| Omy_RAD42793-59 | biallelic SNP | 2 | 0.317 | 0.287 | 0.081 |
| Omy_RAD43573-37 | biallelic SNP | 2 | 0.432 | 0.423 | 0.019 |
| Omy_RAD43694-41 | biallelic SNP | 1.93 | 0.169 | 0.142 | 0.036 |
| Omy_RAD45104-18 | biallelic SNP | 2 | 0.472 | 0.451 | 0.02 |
| Omy_RAD46314-35 | biallelic SNP | 1.93 | 0.123 | 0.112 | 0.046 |
| Omy_RAD46452-51 | biallelic SNP | 2 | 0.471 | 0.478 | 0.016 |
| Omy_RAD46672-27 | biallelic SNP | 2 | 0.468 | 0.438 | 0.03 |
| Omy_RAD47955-51 | biallelic SNP | 2 | 0.293 | 0.252 | 0.019 |
| Omy_RAD4848-14 | biallelic SNP | 1.57 | 0.06 | 0.058 | 0.039 |
| Omy_RAD48799-69 | biallelic SNP | 2 | 0.272 | 0.255 | 0.035 |
| Omy_RAD50632-21 | biallelic SNP | 2 | 0.193 | 0.187 | 0.016 |
| Omy_RAD55404-54 | biallelic SNP | 1.73 | 0.122 | 0.115 | 0.08 |
| Omy_RAD55997-10 | biallelic SNP | 1.14 | 0.002 | 0.002 | -0 |
| Omy_RAD57916-29 | biallelic SNP | 2 | 0.5 | 0.428 | 0.022 |
| Omy_RAD59758-41 | biallelic SNP | 1.59 | 0.037 | 0.037 | 0.019 |
| Omy_RAD59950-44 | biallelic SNP | 1.93 | 0.175 | 0.176 | 0.027 |
| Omy_RAD60135-12 | biallelic SNP | 2 | 0.26 | 0.25 | 0.041 |
| Omy_RAD619-59 | biallelic SNP | 2 | 0.392 | 0.383 | 0.02 |
| Omy_RAD62596-38 | biallelic SNP | 2 | 0.488 | 0.457 | 0.015 |
| Omy_RAD65808-68 | biallelic SNP | 2 | 0.29 | 0.27 | 0.036 |
| Omy_RAD65959-69 | biallelic SNP | 2 | 0.454 | 0.408 | 0.084 |
| Omy_RAD66402-36 | biallelic SNP | 2 | 0.265 | 0.231 | 0.097 |
| Omy_RAD66834-17 | biallelic SNP | 2 | 0.26 | 0.249 | 0.029 |
| Omy_RAD68634-40 | biallelic SNP | 1.61 | 0.104 | 0.099 | 0.048 |
| Omy_RAD739-59 | biallelic SNP | 1.98 | 0.202 | 0.186 | 0.031 |
| Omy_RAD76060-20 | biallelic SNP | 1.43 | 0.072 | 0.038 | 0.067 |
| Omy_RAD77789-54 | biallelic SNP | 2 | 0.32 | 0.316 | 0.026 |
| Omy_RAD78147-27 | biallelic SNP | 2 | 0.44 | 0.415 | 0.033 |
| Omy_RAD78502-57 | biallelic SNP | 1.93 | 0.093 | 0.09 | 0.03 |
| Omy_RAD78776-10 | biallelic SNP | 1.96 | 0.103 | 0.101 | 0.02 |
| Omy_RAD85131-35 | biallelic SNP | 2 | 0.402 | 0.296 | 0.086 |
| Omy_RAD88028-7 | biallelic SNP | 2 | 0.39 | 0.375 | 0.043 |
| Omy_RAD88122-32 | biallelic SNP | 2 | 0.314 | 0.288 | 0.027 |
| Omy_RAD93580-37 | biallelic SNP | 2 | 0.399 | 0.39 | 0.023 |
| Omy_RAD98715-53 | biallelic SNP | 2 | 0.394 | 0.337 | 0.032 |
| Omy_rapd-167 | biallelic SNP | 2 | 0.304 | 0.302 | 0.026 |
| Omy_rbm4b-203 | biallelic SNP | 1.89 | 0.268 | 0.215 | 0.057 |
| Omy_redd1-410 | biallelic SNP | 2 | 0.319 | 0.309 | 0.039 |
| Omy_srp09-37 | biallelic SNP | 2 | 0.428 | 0.427 | 0.022 |
| Omy_sSOD-1 | biallelic SNP | 1.52 | 0.026 | 0.025 | 0.019 |
| Omy_star-206 | biallelic SNP | 1.75 | 0.107 | 0.103 | 0.024 |
| Omy_stat3-273 | biallelic SNP | 2 | 0.348 | 0.34 | 0.03 |
| Omy_tlr3-377 | biallelic SNP | 1.89 | 0.174 | 0.168 | 0.034 |
| Omy_u07-79-166 | biallelic SNP | 1.91 | 0.187 | 0.183 | 0.045 |
| Omy_u09-53.469 | biallelic SNP | 2 | 0.481 | 0.448 | 0.055 |
| Omy_u09-54-311 | biallelic SNP | 2 | 0.43 | 0.429 | 0.022 |
| Omy_u09-61.043 | biallelic SNP | 1.27 | 0.015 | 0.015 | 0.015 |
| Omy_UBA3b | biallelic SNP | 2 | 0.235 | 0.227 | 0.038 |
| Omy_UT16_2-173 | biallelic SNP | 1.96 | 0.114 | 0.107 | 0.02 |
| Omy_vamp5-303 | biallelic SNP | 2 | 0.338 | 0.318 | 0.019 |
| Omy_vatf-406 | biallelic SNP | 2 | 0.481 | 0.457 | 0.071 |
| Omy_zg57-91 | biallelic SNP | 1.89 | 0.171 | 0.163 | 0.04 |
| OMY1011SNP | biallelic SNP | 2 | 0.378 | 0.31 | 0.027 |
